# Dose- and time-dependent effects of hyaluronidase on structural cells and the extracellular matrix of the skin

**DOI:** 10.1186/s40001-020-00460-z

**Published:** 2020-11-23

**Authors:** Bettina Alexandra Buhren, Holger Schrumpf, Katharina Gorges, Oliver Reiners, Edwin Bölke, Jens W. Fischer, Bernhard Homey, Peter Arne Gerber

**Affiliations:** 1 Department of Dermatology, University Hospital Duesseldorf, Duesseldorf, Germany; 2 Department of Pharmacology, University Hospital Duesseldorf, Duesseldorf, Germany; 3 Department of Radiation Oncology, Medical Faculty, University Hospital Duesseldorf, Duesseldorf, Germany; 4 Dermatologie am Luegplatz, Duesseldorf, Germany

**Keywords:** Skin, Dermatology, Metabolism, Enzymes, Cell

## Abstract

**Introduction:**

Hyaluronic acid (hyaluronan; HA) is an essential component of the extracellular matrix (ECM) of the skin. The HA-degrading enzyme hyaluronidase (HYAL) is critically involved in the HA-metabolism. Yet, only little information is available regarding the skin’s HA–HYAL interactions on the molecular and cellular levels.

**Objective:**

To analyze the dose- and time-dependent molecular and cellular effects of HYAL on structural cells and the HA-metabolism in the skin.

**Materials and methods:**

Chip-based, genome-wide expression analyses (Affymetrix® GeneChip PrimeView™ Human Gene Expression Array), quantitative real-time PCR analyses, enzyme-linked immunosorbent assay (ELISA), immunohistochemistry (DAB), and in vitro wound healing assays were performed to assess dose-dependent and time-kinetic effects of HA and HYAL (bovine hyaluronidase, Hylase “Dessau”) on normal human dermal fibroblasts (NHDF), primary human keratinocytes in vitro and human skin samples ex vivo.

**Results:**

Genome-wide expression analyses revealed an upregulation of HA synthases (HAS) up to 1.8-fold change in HA- and HYAL-treated NHDF. HA and HYAL significantly accelerated wound closure in an in vitro model for cutaneous wound healing. HYAL induced HAS1 and HAS2 mRNA gene expression in NHDF. Interestingly, low concentrations of HYAL (0.015 U/ml) resulted in a significantly higher induction of HAS compared to moderate (0.15 and 1.5 U/ml) and high concentrations (15 U/ml) of HYAL. This observation corresponded to increased concentrations of HA measured by ELISA in conditioned supernatants of HYAL-treated NHDF with the highest concentrations observed for 0.015 U/ml of HYAL. Finally, immunohistochemical analysis of human skin samples incubated with HYAL for up to 48 h ex vivo demonstrated that low concentrations of HYAL (0.015 U/ml) led to a pronounced accumulation of HA, whereas high concentrations of HYAL (15 U/ml) reduced dermal HA-levels.

**Conclusion:**

HYAL is a bioactive enzyme that exerts multiple effects on the HA-metabolism as well as on the structural cells of the skin. Our results indicate that HYAL promotes wound healing and exerts a dose-dependent induction of HA-synthesis in structural cells of the skin. Herein, interestingly the most significant induction of HAS and HA were observed for the lowest concentration of HYAL.

## Introduction

The extracellular matrix (ECM) of the skin is a complex network of macromolecules, and plays an important role in the regulation of numerous cellular mechanisms such as proliferation, adhesion, migration, and gene regulation in addition to their mechanically stabilizing function [5]. A functionally and quantitatively important component of dermal ECM is hyaluronic acid (hyaluronan; HA) [26]. Approximately half of all HA in the body is contained within skin tissue. Due to its hydrophilic properties, HA binds high volumes of water which in turn determines the physical properties of tissues (e.g., the viscoelasticity of the skin) [31]. In contrast to other dermal glycosaminoglycans, the biosynthesis of HA does not take place in the Golgi apparatus, but on the inside of the cell membrane by localized HA synthases (HAS1, HAS2 and HAS3) [11]. The different HAS isoforms produce HA which differs primarily in the polymer size. HAS1 and HAS3 synthesize HA polymers in the order of 2 × 10^5^ to 2 × 10^6^ Da, while HAS2 forms HA polymers > 2 × 10^6^ Da [59]. The half-life of HA is organ-dependent and is approximately 24 h in the skin. The degradation of HA is mediated via free chemical radicals and different hyaluronidases (HYAL1 and HYAL2) first into smaller fragments of different sizes, which are then further degraded [51].

Depending on the fragment size, degradation products also have differing biological properties and may, for example induce neovascularization resulting in a proinflammatory response. The expression of different sized HA fragments and also the degradation of HA to HA fragments of different sizes are thus critically important in the regulation of the ECM [27]. Hence, in addition to its importance as a structural molecule, HA is also considered a functional molecule, depending upon its molecular size [14, 55].

In ophthalmological and surgical applications, HYAL is primarily employed as a so-called spreading factor for cutaneous infiltration, as the addition of HYAL to infiltrating local anesthetics accelerates anesthetic diffusion and expansion of the anesthetized area [40, 58, 60]. In addition to its use in local anesthesia, HYAL is used to manage complications following aesthetic injections of HA-fillers. In aesthetic medicine the injection of HA-based fillers for soft tissue augmentation, deep skin hydration or facial contouring has become increasingly popular over the past decades. Besides overcorrections potential complications of aesthetic HA-fillers include edema, infections, or even skin necrosis or visual complications [6, 22, 25, 58]. As HYAL has the potency to effectively degrade HA-based fillers, the off-label use of HYAL is considered as the gold standard for the management of complications of HA fillers [6]. To date, little information is available regarding the mechanisms of HA catabolism and HYAL–HA interactions at the cellular and molecular levels in the skin. We therefore systematically assessed the molecular and cellular effects of HA and HYAL (Hylase® “Dessau”) on the gene regulation in structural skin cells and evaluated the role of HA and HYAL on the healing of artificial wounds in vitro.

## Materials and methods

### Reagents

The hyaluronan (HA) Juvederm Ultra 3 (Allergan, Dublin, Ireland) has been widely used as an injectable filler in aesthetics dermatology. Its main indication is filling of folds and correction of soft tissue loss due to disease or age [19]. Juvederm Ultra 3 is made of cross-linked HA in a monophasic state and contains HA in a mixture of high-molecular-weight (HMW) polymers of 491 kDa (38%) and low-molecular-weight (LMW) polymers of 134 kDa (62%) [17]. We decided to use the dose of 1 mg/ml as this concentration turned out to be optimal in our preliminary experiments, especially with regard to handling (viscosity, etc.). In addition, this specific concentration has been widely used and published in previous studies [10, 24].

For the hyaluronidase (HYAL) Hylase “Dessau” (Riemser, Greifswald, Germany), we decided to use tenfold serial dilutions allowing us to compare a wide range of doses. This is a common method for such dose-range findings, the dose-by-factor approach [39, 48]. The stock concentration of Hylase “Dessau” was 150 U/ml. This value was divided multiple times by 10 in order to obtain the following concentrations in “International Units”: 15 U/ml, 1.5 U/ml, 0.15 U/ml and 0.015 U/ml.

### Cell culture

All research involving human samples was approved by the Medical Faculty of the University of Duesseldorf. Written informed consent was obtained from each participant.

The commercially available normal human dermal fibroblasts (NHDF) were isolated from the dermis of juvenile foreskin (PromoCell, Heidelberg, Germany) and handled according to the manufacturer’s instructions. Briefly, for experimental setup NHDF were cultured in 6-well plates at 37 °C in 5% CO_2_ in cell-specific medium Quantum 333 (PAA, Pasching, Austria) supplemented with 2 mM l-glutamate, 100 U/ml penicillin, and 100 µg/ml streptomycin. When the cells reached approximately 80% of confluency (80% of surface of flask covered by cell monolayer) they were used for experiments.

The primary human keratinocytes were used as described elsewhere [33]. In more detail, primary human keratinocytes were isolated from non-sun-exposed adult skin (age ranged from 35 to 60 years; mean age was 47). After fat and loose fascia were trimmed, skin fragments were placed into 50 ml tubes at 4 °C overnight for dispase digestion (1.5 U/ml; GIBCO, Invitrogen, Carlsbad, USA). The epidermal pieces were transferred to another tube containing 2 ml 0.05% trypsin/EDTA solution (Merck, Darmstadt, Germany) and were incubated for about 30 min. Following neutralization, the cell suspension of epidermal cells was filtered and finally released into keratinocyte-SFM medium (ThermoFisher, Waltham, MA), supplemented with recombinant EGF, pituitary extract, 2 mM l-glutamate, 100 U/ml penicillin, and 100 µg/ml streptomycin. Cells were then cultured at 37 °C and 5% CO_2_ in 6-well plates until cells reached approximately 80% of confluency or cryopreserved until further use.

The number of different individual donors was *n* ≤ 6 for keratinocytes. The age of donors ranged from 35 to 60 years, the mean age was 47. For fibroblasts, the number of different independent experiments was *n* = 4.

Primary cells were treated with 1 mg/ml HA Juvederm Ultra 3 and/or HYAL Hylase “Dessau” for different incubation time points (0 h, 4 h, 12 h, 24 h) and different enzyme doses (15 U/ml, 1.5 U/ml, 0.15 U/ml, 0.015 U/ml).

For investigation of the Affymetrix®-based genome-wide expression analysis, cells were treated with 1 mg/ml Juvederm Ultra 3 HA and/or 1.5 U/ml HYAL for 24 h.

### RNA extraction

RNA from primary human keratinocytes and NHDF was isolated for expression analyzes using RNeasy Mini Kit (Qiagen, Hilden, Germany) according to the manufacturer's protocol. The yield of RNA was determined using a NanoDrop™ 2000c photometer (ThermoFisher, Waltham, MA). A value between 1.8 and 2.1 for the OD 260/280 [optical density (OD) ratio at a wavelength of 260/280 nm] indicated that the extracted RNA contained no interfering proteins, salts or other contaminants. The quality of RNA obtained was subsequently checked bioanalytically (Agilent® Bioanalyzer assay RNA 6000 Pico Chip™, Santa Clara, CA).

### Microarray hybridization

For the assessment of gene regulation by means of Affymetrix® chip-based, genome-wide expression analysis the hybridization of purified and bioanalytically immaculate RNA [RNA Integrity Number (RIN) > 9] from NHDF was carried out according to the manufacturer's instructions, followed by statistical analysis. Background adjustment, signal normalization, and summarization were performed using the Robust Multi-array Average (RMA) algorithm in ArrayAssist™ software (Iobion Labs, La Jolla, CA). Raw data, filtered by expression (20th to 100th percentile), were output as fold change (≥ ± 1.5). Untreated (medium only) NHDF were used as controls.

### Quantitative real-time PCR analysis

Quantitative real-time PCR analysis was performed as described by Homey and colleagues [23]. RNA from both primary human keratinocytes and NHDF was treated with DNase I (Roche, Basel, Switzerland) and reverse transcribed with Oligo(dT)12–18 (ThermoFisher, Waltham, MA) and random hexamer primers (Promega, Madison, WI) using standard protocols. cDNA was analyzed for the expression of human HAS1, HAS2 and HAS3 genes using a QuantStudio™ 6 Flex Real-Time PCR System (ThermoFisher, Waltham, MA). cDNA was amplified in the presence of SYBR™ Green master mix (ThermoFisher, Waltham, MA), gene-specific forward and reverse primers, and water. Primers were obtained from Eurofins Genomics (Ebersberg): HAS1 forward 5′-TCGGAGATTCGGTGGACTA-3′, reverse 5′-AGGAGTCCAGAGGGTTAAGGA-3′, HAS2 forward 5′-GTGGATTATGTACAGGTTTGTGA-3′, reverse 5′-TCCAACCATGGGATCTTCTT-3′, HAS3 forward 5′-CGATTCGGTGGACTACATCC-3′, reverse 5′-CCTACTTGGGGATCCTCCTC-3′. Target gene expression was normalized to the expression of 18S rRNA.

### Cutaneous wound healing assay

Tissue regeneration is quite a complex process that consists of a sequence of events including inflammation, proliferation, and migration of different cells like fibroblasts [4]. There are a number of human in vitro models available which include different levels of complexity. In line with the 3Rs (reduction, refinement and replacement of test animals), we investigated cell mobility during wound healing in a scratch wound healing assay [38]. In our analyses this assay was established on a monolayer of normal dermal human fibroblasts to study random fibroblast migration towards different treatment conditions.

Therefore, NHDF were cultured in 12-well plates until 95% confluency. Cells were treated as previously described. In addition, NHDF treated with medium-sized HA (Hyaluronan (Medium MW), R&D Systems, Minneapolis, USA) with a fragment size from 75 to 350 kDa were used. The monolayer of cells was scratched across each well using a fine pipette tip in order to create a cell-free area. The condition of scratches was detected from time point 0 using a digital time lapse video system (Zeiss® Axiovert™ 200M and AxioVision™ software 4.7, Oberkochen, Germany) over a period of 50 h. The evaluation of end-point assays was carried out by comparing the wound closure of the control with the wound healing response of cells treated with HA and/or HYAL using the program TScratch (CSElab, Zurich, Switzerland).

### Enzyme-linked immunosorbent assay (ELISA)

HA concentrations in the supernatants of HA- and/or HYAL-stimulated primary human keratinocytes and NHDF were measured using an enzyme-linked immunosorbent assay (DuoSet® ELISA, R&D Systems, Minneapolis, USA).

This assay was performed according to the manufacturer’s instructions and is able to detect the low-molecular weight (15–40 kDa), medium molecular weight (75–350 kDa), and high molecular weight (> 950 kDa) forms of hyaluronan.

Briefly, monoclonal capture antibody was incubated overnight in the wells of an immunosorbent 96-well plate. After blocking with reagent diluents (1% BSA in PBS) for 1 h at room temperature, wells were aspirated and rinsed with washing buffer (0.05% Tween® 20 in PBS). Following another aspiration and washing step, biotinylated detection antibody was incubated for 2 h. After next aspiration and washing step, streptavidin-HRP was incubated for 20 min. Following a final aspiration and washing step, substrate solution was incubated for 20 min. Finally, stop solution was added. Optical densities were measured at 450 nm by using a microplate reader. Sample concentrations were calculated against standard curves.

### Skin organ cultures

Human skin bunch biopsies, isolated from non-sun-exposed adult skin (age ranged from 35 to 60 years), were obtained from individuals following elective surgery with full ethical approval and informed consent. The skin samples were processed to remove the underlying fat and connective tissue. Ex vivo skin samples were cultured at the air–liquid interface with the epidermal side up 48 h in keratinocyte-SFM (ThermoFisher, Waltham, MA) supplemented with recombinant EGF and stimulated for 24 h at 37 °C as mentioned above, followed by washings three times for 5 min with phosphate-buffered saline (PBS). Thereafter ex vivo skin samples were fixed with 10% buffered formalin, and embedded in paraffin wax before performing 10-µm cross skin sections.

### Immunohistochemistry (DAB) on paraffin-mounted normal skin tissue slides

Heat-fixed paraffin-mounted normal skin slides were deparaffinized three times with Roticlear® I, II, III (Roth AG, Arlesheim, Switzerland) for 15 min per treatment, then hydration once each to 100%, 95%, and 70% ethanol for 2 min, followed by washings with PBS. Slides were subjected to immunohistochemistry by using a DAB staining kit (Vector Laboratories, Burlingame, CA). Briefly, the slides were blocked for 20 min using an avidin/biotin blocking kit (Vector Laboratories, Burlingame, CA), followed by washing with PBS. Then, slides were blocked for 30 min with 1% BSA/10% FCS in TBS followed by incubation with a biotinylated HA binding protein (Merck Chemicals GmbH, Darmstadt, Germany) (1:200) in 1% BSA overnight at 4 °C. After washings with PBS and blocking with 3% H_2_O_2_ in between, slides were then rinsed with PBS and incubated with secondary antibody for 1 h at room temperature. The slides were washed again and developed with 3,3′-diaminobenzidine (DAB) as substrate according to the manufacturer's instructions. Subsequently, a nuclear staining with hemalum was performed. The slides were mounted with Roti®-Mount (Roth AG, Arlesheim, Switzerland). For quantification of DAB staining, slides were photographed by a Zeiss® Axiovert™ 200M microscope and AxioVision™ software 4.7 (Oberkochen, Germany). Next, DAB staining was analyzed by ImageJ software (BioVoxxel Fiji ImageJ 1.49 m). Values were normalized and represented as positive staining per area in relative units.

### Statistical analysis

Data were expressed at mean ± standard error of the mean (SEM). Statistical significance was assessed by Student’s *t*-test. *P*-values less than or equal to 0.05 were considered statistically significant (*p ≤ 0.05, **p ≤ 0.01, ***p ≤ 0.001).

## Results

### HYAL and HA induce HAS expression in NHDF in vitro

**Fig. 1:**
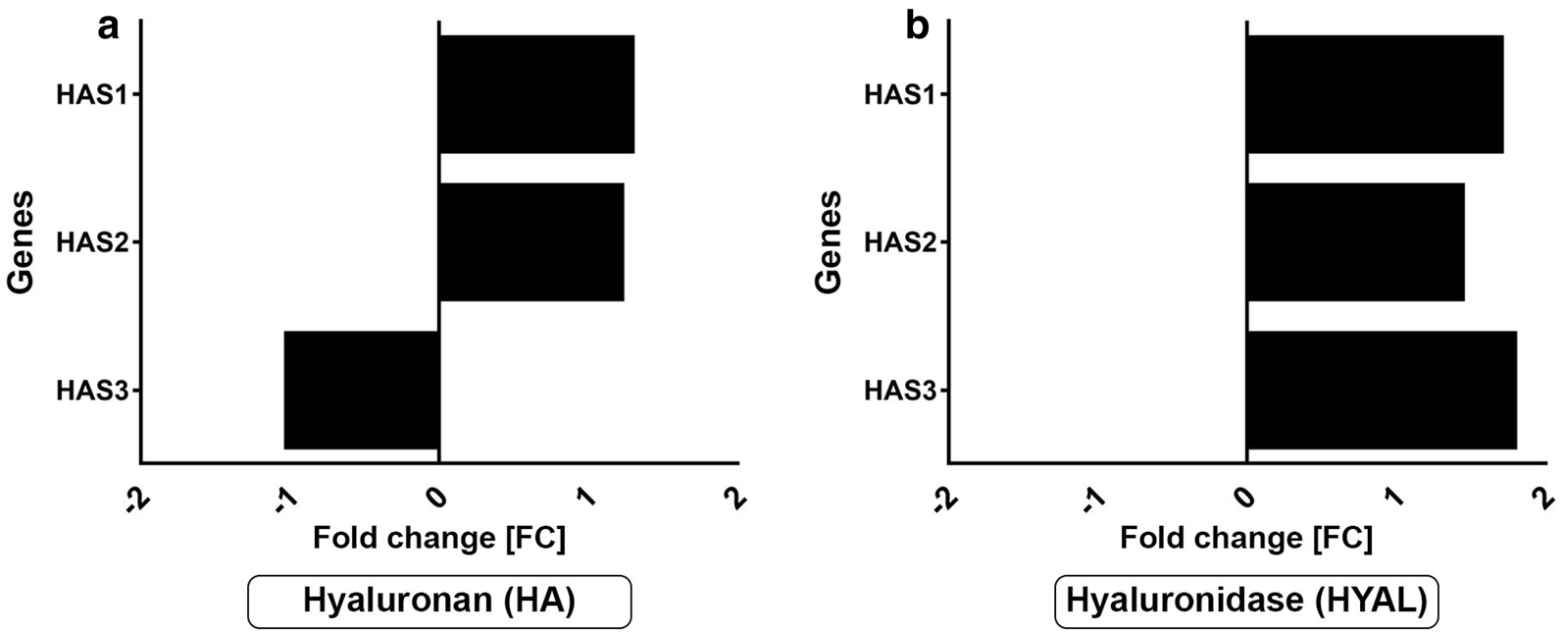
Hyaluronan (HA) and hyaluronidase (HYAL) induce the mRNA expression of HA synthases. Affymetrix® GeneChip expression data (*n* = 1) of **a** HA- and **b** HYAL-treated NHDF shown as fold changes [FC]

Affymetrix® expression analyses were carried out to systematically investigate the effects of HA and HYAL in NHDF. Subsequently, in comprehensive bioinformatic analyses, gene lists containing the 50 most upregulated and most downregulated genes were generated (Additional file 1: Tables S1–S6). In NHDF HAS1 and HAS2, transcription level increased 1.2-fold after stimulation with HA. In contrast, HA stimulation decreased gene expression of HAS3 (Fig. 1a). Interestingly, in HYAL-treated NHDF transcription levels of all three HASs increased up to 1.8-fold changes (Fig. 1b).

### HYAL and HA induce HAS in a time- and dose-dependent manner in vitro

To analyze time-kinetic and dose-dependent effects, NHDF and primary human keratinocytes were stimulated with HA and HYAL for different time periods (2 h, 4 h, 12 h and 24 h) as well as different concentrations (15 U/ml, 1.5 U/ml, 0.15 U/ml, 0.015 U/ml).

**Fig. 2:**
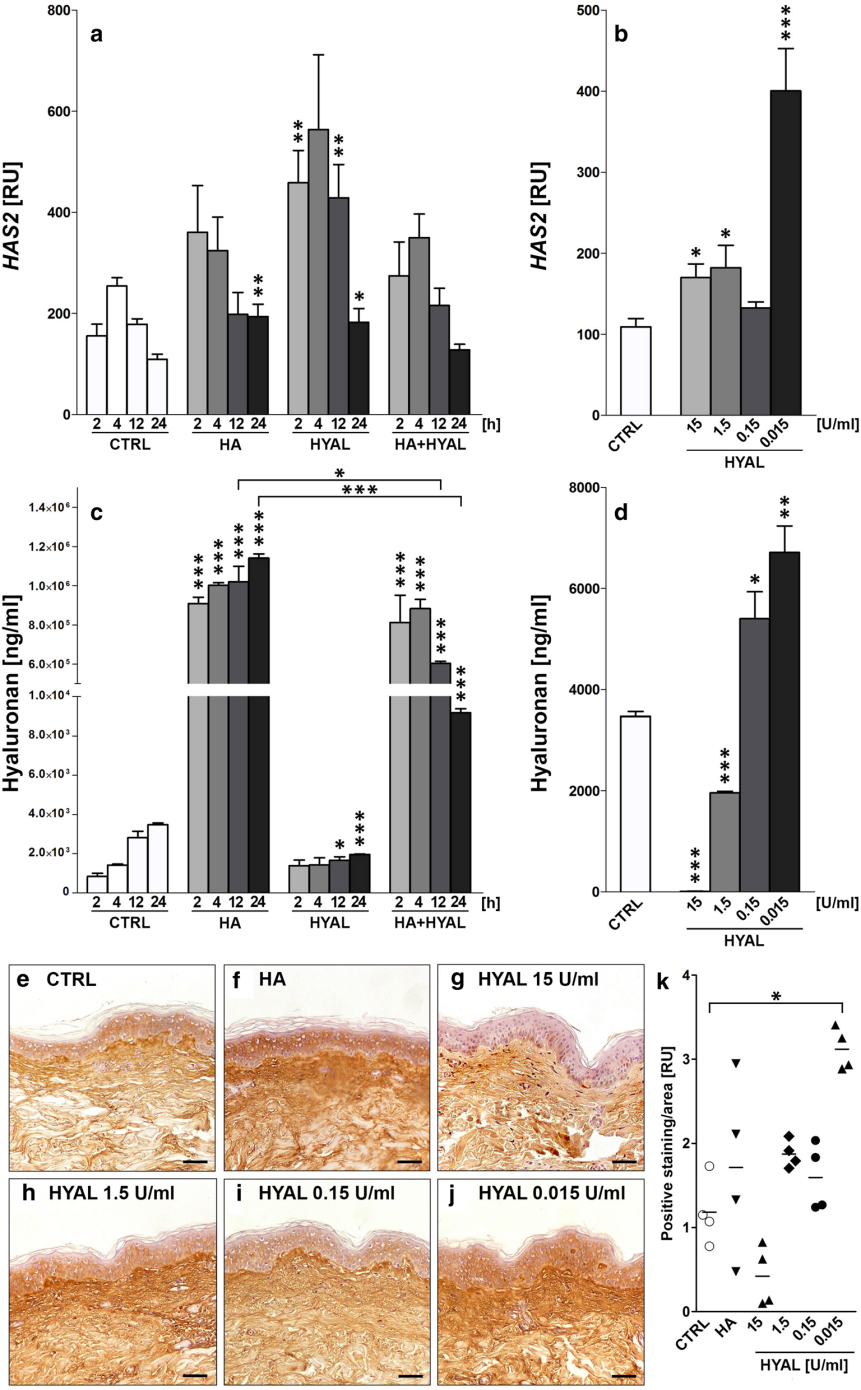
**a** HAS2 gene expression levels (*n* = 4) in normal human dermal fibroblasts (NHDF) after stimulation with 1 mg/ml HA, 1.5 U/ml HYAL and HA + HYAL co-stimulation for 2 h, 4 h, 12 h and 24 h; **b** HAS2 gene expression levels of NHDF after stimulation with 15 U/ml, 1.5 U/ml, 0.15 U/ml and 0.015 U/ml HYAL for 24 h. **c**, **d** HA amount (ng/ml) measurement by means of ELISA (*n* = 4) in supernatants of NHDF treated as described in **a** and **b**. **e**–**k** Show representative histological HA-stained sections of human skin samples treated with **e** control (CTRL) medium, **f** 1 mg/ml HA, **g** 15 U/ml HYAL, **h** 1.5 U/ml HYAL, **i** 0.15 U/ml HYAL and **j** 0.015 U/ml HYAL, scale bars = 50 µm. **k** Quantification of HA-positive staining measured in CTRL, HA and HYAL (15 U/ml, 1.5 U/ml, 0.15 U/ml and 0.015 U/ml) treated skin samples plotted as individual values of *n* = 4, mean values are shown by the horizontal bar. Asterisks above columns indicate statistical significant differences compared to their respective medium controls, *p ≤ 0.05, **p ≤ 0.01, ***p ≤ 0.001 (*t*-test, two-sided)

Stimulation with HA as well as HYAL (1.5 U/ml) for 24 h significantly increased gene expression of HAS2 in NHDF compared to medium controls (Fig. 2a, *p* = 0.0090; *p* = 0.0319). In addition, HYAL treatment for 2 h and 12 h significantly increased gene expression of HAS2 compared to respective medium controls (Fig. 2a, *p* = 0.0012; *p* = 0.0038) with no observed effect for HA. Co-stimulation of HA and HYAL (1.5 U/ml) had no impact on HA synthase gene expression compared to medium control (Fig. 2a, Additional file 1: Figure S1A, C). In contrast, HAS1 expression was significantly induced by HA after 2 h compared to medium controls (Additional file 1: Figure S1A, *p* = 0.0401). Incubation with HYAL (1.5 U/ml) increased gene expression of HAS1 at earlier time points (2 h, 4 h) (Additional file 1: Figure S1A, *p* = 0.0026; *p* = 0.0246). The gene expression profile of HAS3 demonstrated no significant differential regulation when NHDF were treated with HA and/or HYAL (Additional file 1: Figure S1C). In contrast, human epidermal keratinocytes (HEK) were less responsive to HA and HYAL with regard to HAS1 and HAS2 relative gene expression levels compared to NHDF (Additional file 1: Figure S2A–D). Expression of HAS1 was significantly downregulated at 24 h after stimulation with HA (*p* = 0.0062), HYAL (1.5 U/ml) (*p* = 0.0021) and co-stimulation of HA and HYAL (*p* = 0.0023) as compared to medium control (Additional file 1: Figure S2A). At early time points (2 h, 4 h) co-stimulation with HA and HYAL showed significant downregulation of HAS3 (Additional file 1: Figure S2E, *p* = 0.0317; *p* = 0.0032).

Next, different doses of HYAL were tested in NHDF. Interestingly, HAS2 expression increased with decreasing concentrations of HYAL (Fig. 2b). Notably, the lowest tested concentration of HYAL (0.015 U/ml) demonstrated a highly significant induction of HAS2 expression compared to medium control (Fig. 2b, *p* = 0.0002). Similarly, incubation with HYAL at its lowest concentration also induced gene expression of HAS1 (Additional file 1: Figure S1B, *p* = 0.0106). Gene expression of HAS3 was not affected when NHDF were stimulated with different doses of HYAL (Additional file 1: Figure S1D). Varying doses of HYAL were then tested in primary human keratinocytes. In contrast, stimulation with HYAL significantly decreased expression of HAS1 (Additional file 1: Figure S2B) while HAS2 and HAS3 were not affected by varying doses of HYAL for 24 h (Additional file 1: Figure S2D, F).

### HYAL induces HA production in NHDF but not in HEK in a time- and dose-dependent manner in vitro

To analyze soluble HA release, conditioned supernatants of time- and dose-dependent experiments (see above) in NHDF and primary human keratinocytes were analyzed by ELISA. HA secretion increased continuously over time in medium control (Fig. 2c, Additional file 1: Figure S1G). As expected, the addition of HA to primary cells resulted in a higher concentration of HA. Treatment with HYAL (1.5 U/ml) reduced HA concentration at 12 h (*p* = 0.0209) and 24 h (*p* < 0.0001) compared to medium controls in NHDF. Co-stimulation with HYAL and HA decreased HA-concentration over time compared to stimulation with HA only. Next, supernatants of cells stimulated with varying HYAL concentrations were analyzed. Interestingly, while the incubation with higher concentrations of HYAL (15 U/ml and 1.5 U/ml) showed significantly lower concentrations of HA (Fig. 2d, *p* < 0.0001; *p* < 0.0001), treatment with HYAL at lower concentrations (0.15 U/ml and 0.015 U/ml) significantly increased the concentration of HA when compared to medium controls in NHDF (Fig. 2d, *p* = 0.0286; *p* = 0.0035). Similar to NHDF, the concentration of HA in supernatants of keratinocytes also increased over time in medium-treated controls (Additional file 1: Figure S2G). The addition of HA increased HA-concentrations in supernatants, which was only marginally reduced in co-stimulated cells. Compared to medium controls, HA concentrations decreased in HYAL (1.5 U/ml) treated keratinocytes at all tested time points (2 h, 4 h, 12 h, 24 h). In contrast to NHDF, the stimulation with different doses of HYAL significantly reduced HA concentrations for tested doses (1.5 U/ml, 0.15 U/ml and 0.015 U/ml) compared to medium controls (Additional file 1: Figure S2H, *p* = 0.0001, *p* = 0.0001, *p* = 0.0005).

### HYAL induces HA in full-thickness human skin samples in a time- and dose-dependent manner ex vivo

Full-thickness human skin samples were treated with HA as well as different doses of HYAL (15 U/ml, 1.5 U/ml, 0.15 U/ml, 0.015 U/ml) ex vivo. Following paraffin embedding and sectioning, skin sections were stained with a biotinylated HA-binding protein to visualize accumulation of HA in the skin by immunohistochemistry (Fig. 2e–j). Computer-assisted quantification of staining intensities showed an induction of HA in HA-treated samples as compared to medium controls (Fig. 2k). Of note, incubation with HYAL at the lowest concentration (0.015 U/ml) resulted in a significantly stronger staining intensity of HA as compared to medium controls (Fig. 2k, *p* = 0.0286).

### HA and HYAL promote wound healing in vitro

**Fig. 3:**
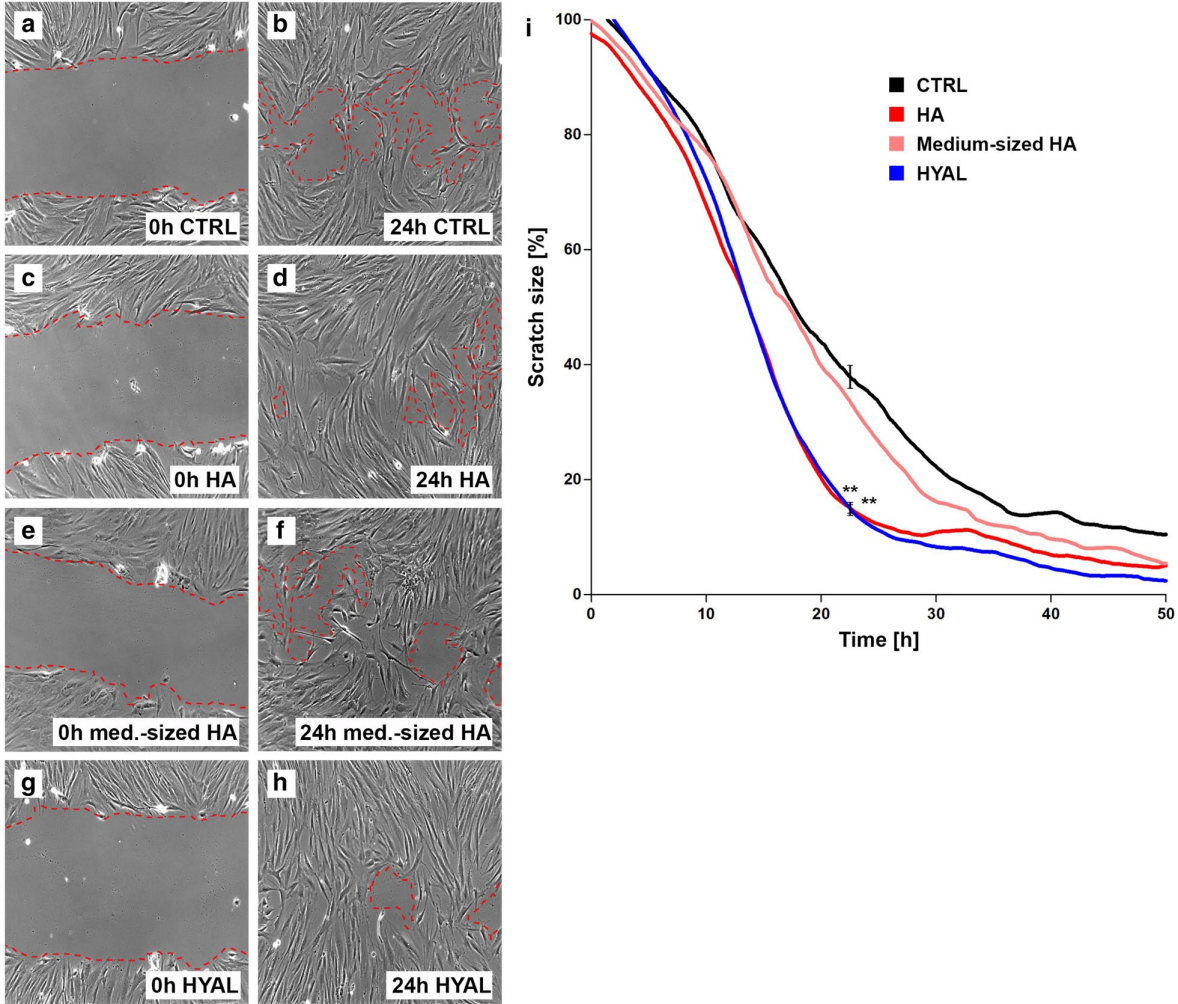
HA and HYAL accelerate wound closure in an in vitro model. Standardized in vitro wound healing model induced by scratching (“wound scratch assay”) a NHDF monolayer of medium control (**a** 0 h, **b** 24 h; black line in **i**), in the presence of HA (1 mg/ml) (**c** 0 h, **d** 24 h; red line in **i**), or medium-sized HA (**e** 0 h, **f** 24 h; pink line in **i**), and HYAL (1.5 U/ml) (**g** 0 h, **h** 24 h; blue line in **i**). The documentation of the wound closure took place over 50 h by means of time-lapse video microscopy. **a**–**h** Show representative images of computer-assisted quantification of the wound area (dotted red line). Values in **i** show percent of scratch size compared to initial scratch size representing the mean of three independent experiments, *p ≤ 0.05, **p ≤ 0.01, ***p ≤ 0.001 (*t*-test, two-sided) (*n* = 3)

Finally, scratch assays were performed to analyze the effects of HA and HYAL on wound healing in vitro. A NHDF monolayer was used to asses wound healing which comprises fibroblast migration and proliferation. Therefore, monolayers of cells were scratched and thereafter stimulated with HA, medium-sized HA and HYAL. Wound closure of treated monolayers was compared to medium controls over 50 h. Stimulation with HA (Fig. 3c, d) and HYAL (Fig. 3g, h) resulted in significantly accelerated wound healing as compared to medium controls (Fig. 3a, b). At 24 h, 83% (HA: *p* = 0.0036, HYAL: *p* = 0.0058) of the scratch area was closed for HA and HYAL as compared to 60% of wound closure for medium-treated controls (Fig. 3i). No significant differences were found for medium-sized HA (Fig. 3e, f) as compared to medium controls (Fig. 3i).

## Discussion

To date, the effects of HA and HYAL on structural cells of the skin have been poorly characterized. Here, we examined these effects by comprehensive genome-wide gene chip analyzes followed by qPCR validation and quantitative protein analyzes.

Comprehensive literature suggests a predominant role of fibroblasts in HA metabolisms. In previous studies, Röck et al. found that HA is synthesized and incorporated as a quantitative and functionally important component into the dermal ECM [47].

There are a variety of chemical signals known to stimulate HA synthesis in human fibroblasts such as cytokines, decreased pH, growth factors as well as enzymatic degradation of HA [20, 29, 30]. Underlying mechanisms remain unclear. In line with other findings, enzymatic degradation of HA but also HA itself was found to stimulate HA in an in vitro cell culture system. In ^3^H-glucosamine labeling experiments Moczar and Robert found that treatment of human skin fibroblasts with bovine testicular hyaluronidase increased the amount of newly synthesized HA in the medium [37]. In line with these results, our results show that HYAL increased HA amounts in conditioned supernatants of NHDF as measured by ELISA.

Interestingly, increased HA amounts were found particular in supernatants of those cells which showed high gene expression of HAS2 but no other isoforms. In various studies the HAS isoform HAS2 has been suggested to be most important for HA synthesis. HAS2 is the only HAS gene which deletion causes a lethal phenotype: HAS2 knockout mice die at embryonic day (E) 9.5 due to a failure to form HA-rich organs [8]. This confirms the predominant role of HAS2 in the regulation of HA and reveals its important role for HA-metabolism. Moreover, HAS2 appeared to be the predominant isoform in skin fibroblasts, based on the results of the quantitative real-time RT-PCR [47]. In addition Averbeck et al. [2] found that HAS-1 and HAS-2 were much more highly expressed in fibroblasts than in HaCaT and human skin.

However, the increase of HA amount in the supernatants could either result from (i) increase in HA synthesis or (ii) clearing of membrane-bound HA, but also (iii) increase of HA degradation mediated by HYAL. Since HYAL activity was not investigated in our experiments, further studies are required to address this specific question.

**Fig. 4:**
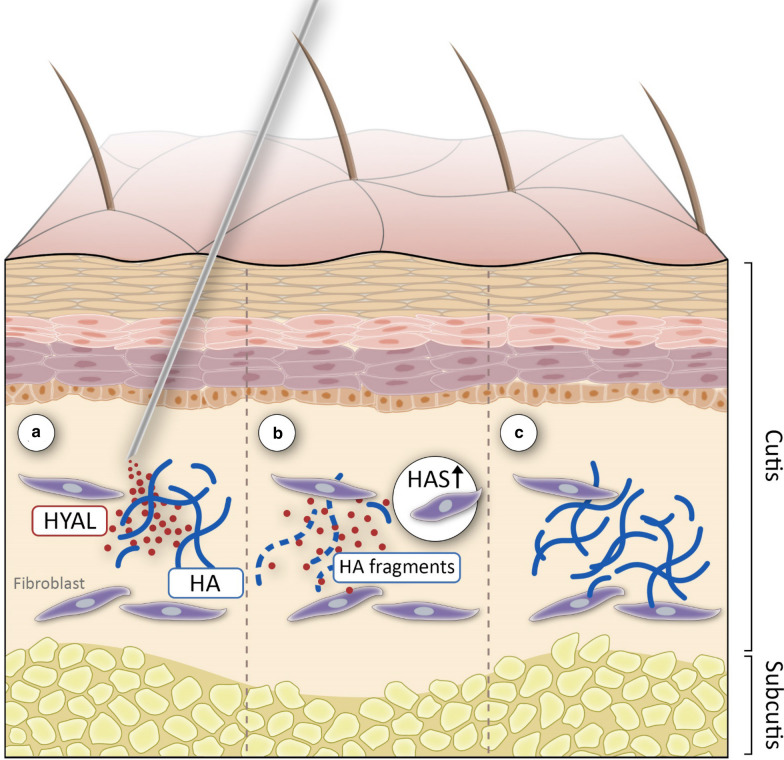
Injection of low-dose HYAL (**a**) degrades HA in the extracellular matrix of the skin (**b**). HYAL and breakdown fragments of HA might be involved in induction of HA synthases resulting in accumulation of HA in the skin (**c**)

In titration experiments we showed that HAS2 gene expression increased with decreasing concentrations of HYAL. Interestingly, HYAL at its lowest concentration (0.015 U/ml) led to the strongest induction of HAS2. Correspondingly, the amount of newly synthesized HA was the highest in cells treated with in low doses of HYAL. Furthermore, immunohistochemical analyses of human skin samples incubated with HYAL ex vivo demonstrated that low concentrations of HYAL (0.015 U/ml) led to a pronounced accumulation of HA, whereas high concentrations of HYAL (15 U/ml) reduced dermal HA levels. In similar observations Philipson et al. [43] found that HYAL treatment at very low concentrations stimulated HA synthesis not only in cultured cells but also in isolated membrane preparations [42] suggesting an existing feedback mechanism that enables cells to sense levels of HA that has been synthesized [49]. The exogenously added HYAL cleaves newly synthesized HA chains as they are being extruded through pore-like structures out of the cell into the extracellular space [44] leaving a message for fibroblasts that insufficient quantities of HA have been synthesized which might result in induced HA synthesis [50]. As early as 1986 Mian postulated the existence of a multi-protein-membrane associated complex that is able to synthesize HA but also has catabolic activity [35, 36]. Two decades later Stern suggested a name for this mini-organelle—the hyaluronasome [49]. Comparable to glycogen granules formed in muscle and liver, the hyaluronasome might respond dynamically to extracellular and intracellular events being able to regulate levels of HA deposition [49]. An organelle in which all components are tethered together (containing HA receptors such as RHAMM and CD44 and HAS but also HYAL and HA-binding proteins) would provide the structural organization for such reactions to occur with maximum efficiency [49, 56]. The existence of a multiplayer like the hyaluronasome could be a reason why HYAL in its lowest concentration is rather able to modulate and stimulate HA-metabolism in a positive feedback loop (see also Fig. 4), compared to high dose HYAL which would rather lead to a total breakdown of all available HA as demonstrated in our ELISA experiments (Fig. 2).

There is a dynamic feedback signaling between HYAL and HAS regulating the net deposition of HA and HA fragments [21, 54, 59]. Out of a variety of cells, dermal fibroblasts are known to synthesize the largest amounts of HA as compared to other cells of the human organism [32]. In line with this observation, in our study NHDF had a higher basal HA production in contrast to epidermal keratinocytes.

The role of HA and HYAL during wound repair is only poorly described. The healing of cutaneous wounds is a complex biological process that can be divided into different phases that overlap in time and space: hemostasis, inflammation, proliferation, and tissue remodeling [18]. Depending on the basis of its molecular weight, HA can produce different effects [13]. At earlier phases of wound healing in vivo, particular high-molecular weight HA increases at the wounding bed to bind fibrinogen which is essential for clot formation [9, 12]. Later on, in the inflammatory stage of wound healing especially low-molecular weight HA accumulates at the wounding site which is in parts generated from high-molecular weight degradation by increasing levels of wound-produced HYAL [12, 15, 41]. These HA fragments then orchestrate specific size-dependent functions [53]. Extensive literature describes that application of exogenous HA can improve wound healing [1, 3, 7, 28]. In the wound healing analyzes presented here, application of HA induced a significant increase in wound closure. Interestingly, scratch closure occurred as fast in the presence of HYAL. In line with these results, Fronza et al. [18] found that not only HA but also HYAL can accelerate wound closure. In contrast to our in vitro based assay using human primary cells their group used an in vivo full-thickness excisional model in Wistar rats. As a HA degrading enzyme HYAL may contribute to the balance between synthesis and deposition of HA and may therefore play a potential role as a healing promoting agent for cutaneous injuries [18]. Decreased wound healing with age is attributed in part to compromised HA metabolism and decreased ability to process HA [34, 52]. In the aged rat skin, studies have found abundance of HMW-HA, perhaps reflecting an inability to generate lower-molecular-size fragments [46]. The lack to generate such small fragments would compromise the wound healing process [3]. Voorhees and Fisher found that the injection of HA-fillers stimulates localized proliferation of fibroblasts in the human skin [45, 57]. These fibroblasts showed a stretched appearance, and expressed high levels of type I procollagen thereby restoring dermal matrix components that are lost in photodamaged skin [16]. When HYAL is added to the wound scratches it might break cross-links in HA which is being extruded in the medium so it behaves like native HA. Possibly, increased concentration of HA fragments resulting from HYAL activity might be important in the wounding process as they stimulate capacity of fibroblast for functional activation. Particularly low-molecular weight HA has been suggested to contribute to wound healing [59]. Therefore, we also investigated the effects of medium-sized HA on wound closure. Surprisingly, medium-sized fragments did not shorten the closure time of the scratch compared to medium control. As other fragment sizes were not investigated in our study, this could be addressed in future studies.

Wohlrab et al. investigated the influence of adjuvant HYAL on wound healing in a placebo-controlled, double-blinded clinical trial. Regarding target parameters like transepidermal water loss, hemovascular perfusion, and complete macroscopic epithelization of the wound his group found no evidence that HYAL retards wound healing [60].

To conclude, HYAL is a bioactive enzyme that exerts multiple effects on the HA-metabolism as well as on the structural cells of the skin. Our study provides direct evidence that especially low doses of HYAL significantly induce HAS and as well as the synthesis and concentration of HA whereas high-dose-HYAL leads to a downmodulation of HA in dermal fibroblasts. Thus, low-dose-HYAL may be beneficial in the rejuvenation of aged skin as it stimulates dermal fibroblasts to increase HA amount. In addition, our study points toward an important role of HYAL in wound healing as HYAL accelerates wound closure in an in vitro wound scratch model of dermal fibroblasts. Future studies are required to further fully elucidate the underlying molecular pathways of HYAL and HA action in the skin.

## Supplementary information

**Additional file 1: Table S1.** Affymetrix® expression analysis of NHDF treated with HA vs. control showing the 50 most upregulated genes (FC = fold change). **Table S2.** Affymetrix® expression analysis of NHDF treated with HA vs. control showing the 50 most downregulated genes (FC = fold change). **Table S3.** Affymetrix® expression analysis of NHDF treated with medium-sized HA vs. control showing the 50 most upregulated genes (FC = fold change). **Table S4.** Affymetrix® expression analysis of NHDF treated with medium-sized HA vs. control showing the 50 most downregulated genes (FC = fold change). **Table S5.** Affymetrix® expression analysis of NHDF treated with HYAL vs. control showing the 50 most upregulated genes (FC = fold change). **Table S6.** Affymetrix® expression analysis of NHDF treated with HYAL vs. control showing the 50 most downregulated genes (FC = fold change). **Figure S1.** (A, C) HAS1, HAS3 gene expression levels in normal human dermal fibroblasts (NHDF) after stimulation with 1 mg/ml HA, 1.5 U/ml HYAL and HA + HYAL co-stimulation for 2 h, 4 h, 12 h and 24 h, (B, D) HAS1, HAS3 gene expression levels of NHDF after stimulation with 15 U/ml, 1.5 U/ml, 0.15 U/ml and 0.015 U/ml HYAL for 24 h. Asterisks above columns indicate statistical significant differences compared to their respective medium controls. *p ≤ 0.05, **p ≤ 0.01, ***p ≤ 0.001 (*t*-test, two-sided). **Figure S2.** (A, C, E) HAS1, HAS2, HAS3 gene expression levels in primary human keratinocytes after stimulation with 1 mg/ml HA, 1.5 U/ml HYAL and HA + HYAL co-stimulation for 2 h, 4 h, 12 h and 24 h, (B, D, F) HAS1, HAS2, HAS3 gene expression levels in keratinocytes after stimulation with 15 U/ml, 1.5 U/ml, 0.15 U/ml and 0.015 U/ml HYAL for 24 h, (G, H) HA amount (ng/ml) measurement by means of ELISA in supernatants of NHDF treated as described in A–F. Asterisks above columns indicate statistical significant differences compared to their respective medium controls. *p ≤ 0.05, **p ≤ 0.01, ***p ≤ 0.001 (*t*-test, two-sided).

## Data Availability

All data and materials can be accessed via BB and PAG.
