# Supplementary material for: Dose- and time-dependent effects of hyaluronidase on structural cells and the extracellular matrix of the skin
Source: Eur J Med Res. 2020 Nov 23;25:60. doi: 10.1186/s40001-020-00460-z (PMC7686775; doi:10.1186/s40001-020-00460-z)
Supplement: Supplementary file 1 — Additional file 1: Table S1. Affymetrix® expression analysis of NHDF treated with HA vs. control showing the 50 most upregulated genes (FC = fold change). Table S2. Affymetrix® expression analysis of NHDF treated with HA vs. control showing the 50 most downregulated genes (FC = fold change). Table S3. Affymetrix® expression analysis of NHDF treated with medium-sized HA vs. control showing the 50 most upregulated genes (FC = fold change). Table S4. Affymetrix® expression analysis of NHDF treated with medium-sized HA vs. control showing the 50 most downregulated genes (FC = fold change). Table S5. Affymetrix® expression analysis of NHDF treated with HYAL vs. control showing the 50 most upregulated genes (FC = fold change). Table S6. Affymetrix® expression analysis of NHDF treated with HYAL vs. control showing the 50 most downregulated genes (FC = fold change). Figure S1. (A, C) HAS1, HAS3 gene expression levels in normal human dermal fibroblasts (NHDF) after stimulation with 1 mg/ml HA, 1.5 U/ml HYAL and HA + HYAL co-stimulation for 2 h, 4 h, 12 h and 24 h, (B, D) HAS1, HAS3 gene expression levels of NHDF after stimulation with 15 U/ml, 1.5 U/ml, 0.15 U/ml and 0.015 U/ml HYAL for 24 h. Asterisks above columns indicate statistical significant differences compared to their respective medium controls. *p ≤ 0.05, **p ≤ 0.01, ***p ≤ 0.001 (t-test, two-sided). Figure S2. (A, C, E) HAS1, HAS2, HAS3 gene expression levels in primary human keratinocytes after stimulation with 1 mg/ml HA, 1.5 U/ml HYAL and HA + HYAL co-stimulation for 2 h, 4 h, 12 h and 24 h, (B, D, F) HAS1, HAS2, HAS3 gene expression levels in keratinocytes after stimulation with 15 U/ml, 1.5 U/ml, 0.15 U/ml and 0.015 U/ml HYAL for 24 h, (G, H) HA amount (ng/ml) measurement by means of ELISA in supernatants of NHDF treated as described in A–F. Asterisks above columns indicate statistical significant differences compared to their respective medium controls. *p ≤ 0.05, **p ≤ 0.01, ***p ≤ 0.001 (t-test, two-sided [file 40001_2020_460_MOESM1_ESM.docx]

**Additional file 1**

**Table S1.** **Affymetrix^®^ expression analysis of NHDF treated with HA vs. control** showing the 50 most upregulated genes (FC = fold change).

**Table S2.** **Affymetrix^®^ expression analysis of NHDF treated with HA vs. control** showing the 50 most downregulated genes (FC = fold change).

**Table S3.** **Affymetrix^®^ expression analysis of NHDF treated with medium-sized HA vs. control** showing the 50 most upregulated genes (FC = fold change).

**Table S4.** **Affymetrix^®^ expression analysis of NHDF treated with medium-sized HA vs. control** showing the 50 most downregulated genes (FC = fold change).

**Table S5.** **Affymetrix^®^ expression analysis of NHDF treated with HYAL vs. control** showing the 50 most upregulated genes (FC = fold change).

**Table S6.** **Affymetrix^®^ expression analysis of NHDF treated with HYAL vs. control** showing the 50 most downregulated genes (FC = fold change).

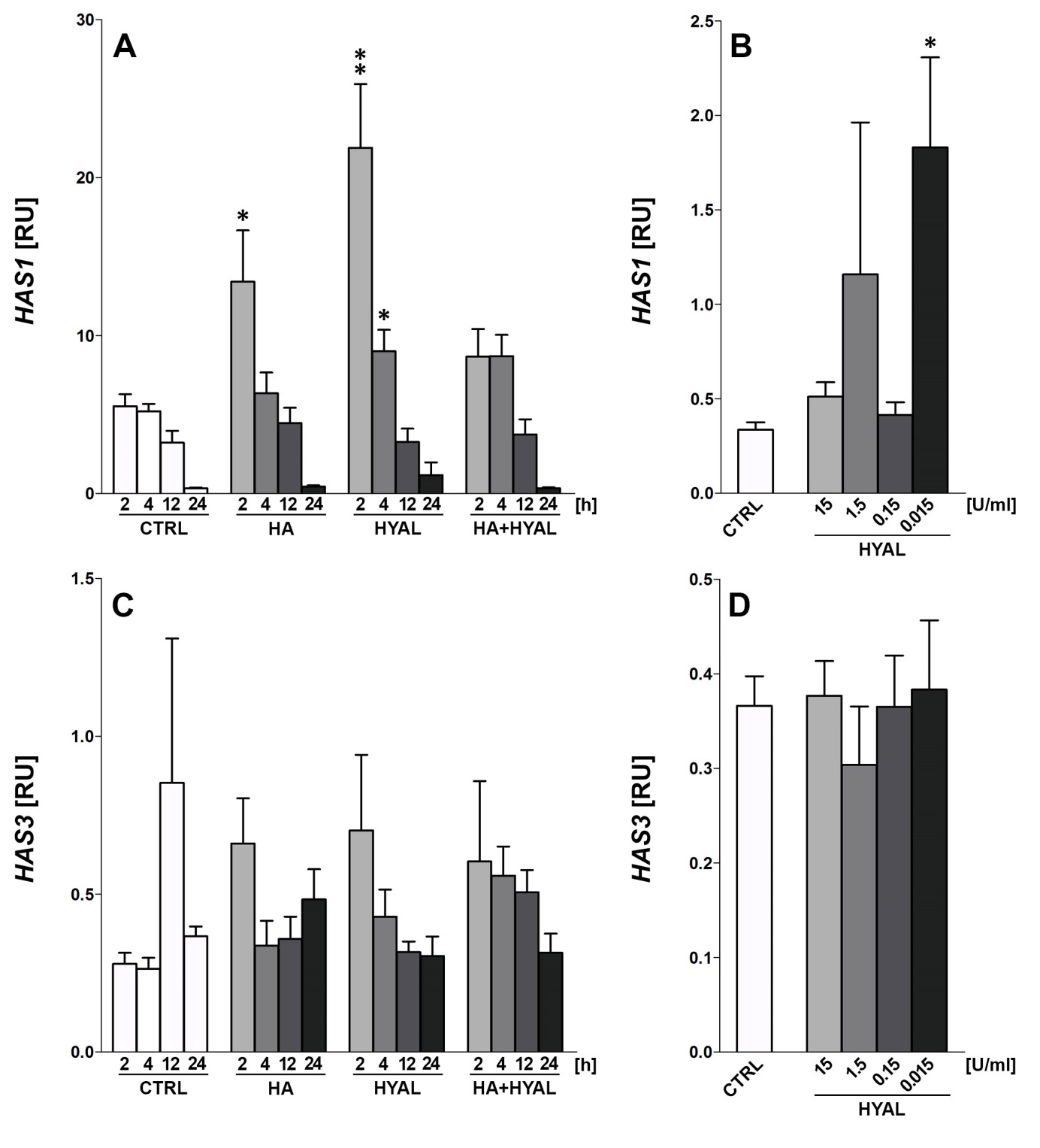
**Figure S1.** (A, C) HAS1, HAS3 gene expression levels in normal human dermal fibroblasts (NHDF) after stimulation with 1 mg/ml HA, 1.5 U/ml HYAL and HA+HYAL co-stimulation for 2 h, 4 h, 12 h and 24 h, (B, D) HAS1, HAS3 gene expression levels of NHDF after stimulation with 15 U/ml, 1.5 U/ml, 0.15 U/ml and 0.015 U/ml HYAL for 24 h. Asterisks above columns indicate statistical significant differences compared to their respective medium controls. *p ≤ 0.05, **p ≤ 0.01, ***p ≤ 0.001 (t-test, two-sided).


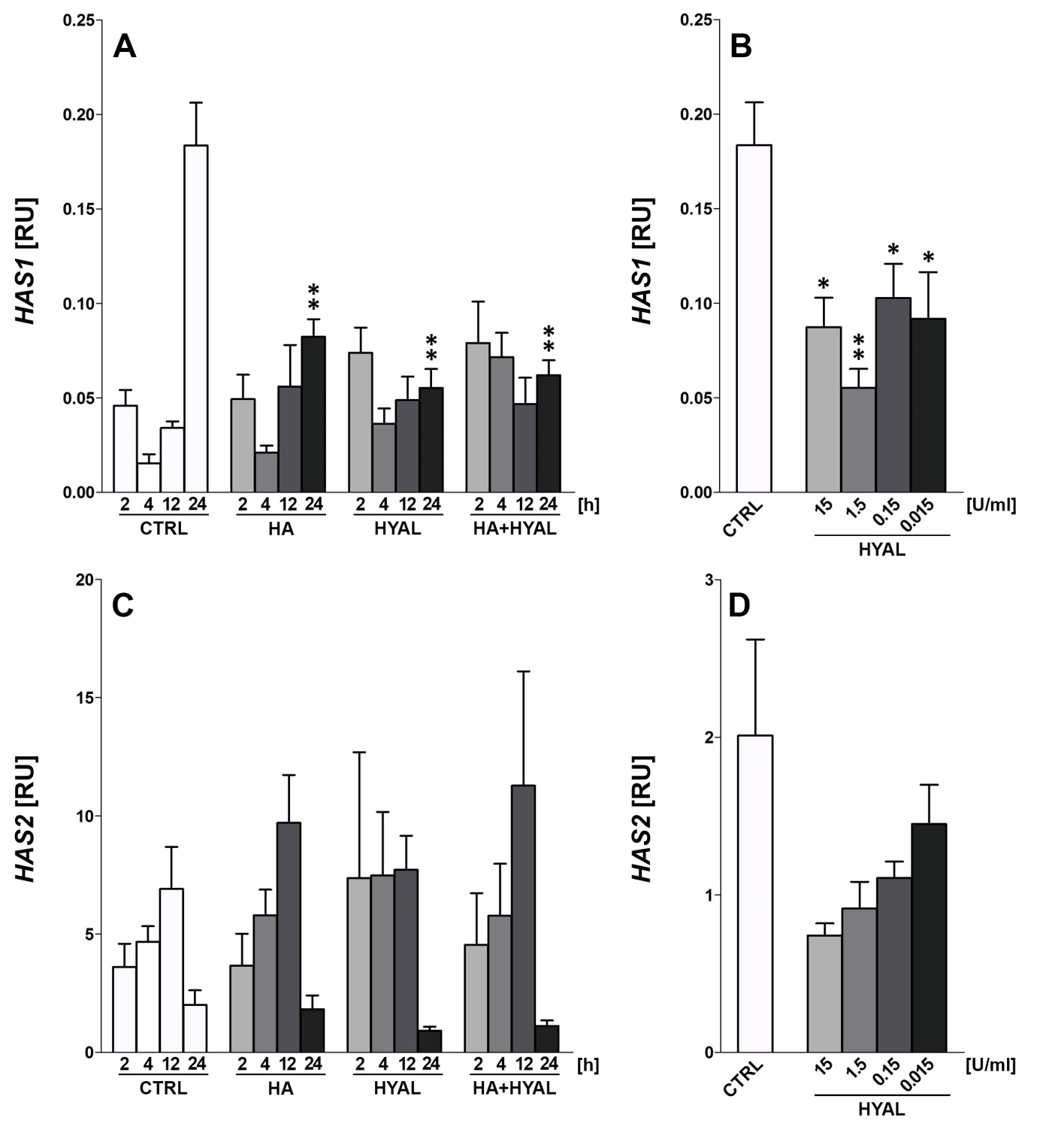


**
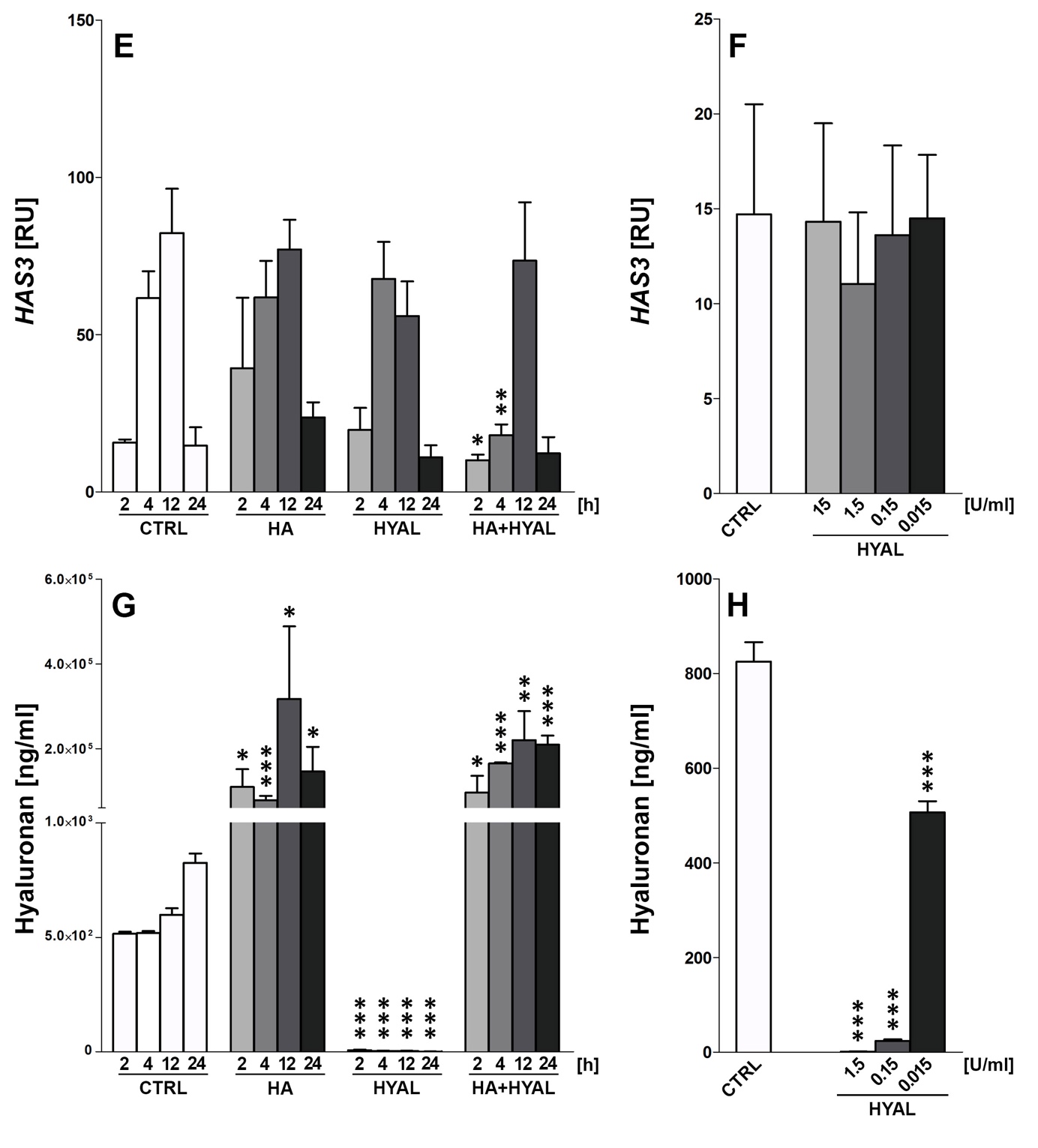
**

**Figure S2.** (A, C, E) HAS1, HAS2, HAS3 gene expression levels in primary human keratinocytes after stimulation with 1 mg/ml HA, 1.5 U/ml HYAL and HA+HYAL co-stimulation for 2 h, 4 h, 12 h and 24 h, (B, D, F) HAS1, HAS2, HAS3 gene expression levels in keratinocytes after stimulation with 15 U/ml, 1.5 U/ml, 0.15 U/ml and 0.015 U/ml HYAL for 24 h, (G, H) HA amount (ng/ml) measurement by means of ELISA in supernatants of NHDF treated as described in A-F. Asterisks above columns indicate statistical significant differences compared to their respective medium controls. *p ≤ 0.05, **p ≤ 0.01, ***p ≤ 0.001 (t-test, two-sided).
